# Influence of antihypertensive drugs on aortic and coronary effects of Ang-(1-7) in pressure-overloaded rats

**DOI:** 10.1590/1414-431X20165520

**Published:** 2017-03-23

**Authors:** A.D.C. Nunes, A.P.S. Souza, L.M. Macedo, P.H. Alves, G.R. Pedrino, D.B. Colugnati, E.P. Mendes, R.A.S. Santos, C.H. Castro

**Affiliations:** 1 Departamento de Ciências Fisiológicas, Universidade Federal de Goiás, Goiânia, GO, Brasil; 2 Instituto Nacional de Ciência e Tecnologia em Nanobiofarmacêutica, Brasil; 3 Departamento de Fisiologia e Biofísica, Universidade Federal de Minas Gerais, Belo Horizonte, MG, Brasil

**Keywords:** Angiotensin-(1-7), Renin-angiotensin system, Coronary artery, Antihypertensive drugs, Cardiac hypertrophy

## Abstract

This study investigated the influence of antihypertensive drugs, such as angiotensin-converting enzyme inhibitors (ACEIs), AT1 receptor blockers (ARBs), voltage-gated L-type calcium channel blockers, and mineralocorticoid receptor antagonists (MRAs), on the effects of angiotensin-(1-7) [Ang-(1-7)] on aorta and coronary arteries from pressure-overloaded rats. Pressure overload was induced by abdominal aortic banding (AB). To evaluate the role of antihypertensive drugs on the effect of Ang-(1-7), AB male Wistar rats weighing 250–300 g were treated with vehicle or low doses (5 mg·kg^-1^·day^-1^, gavage) of losartan, captopril, amlodipine, or spironolactone. Isolated aortic rings and isolated perfused hearts under constant flow were used to evaluate the effect of Ang-(1-7) in thoracic aorta and coronary arteries, respectively. Ang-(1-7) induced a significant relaxation in the aorta of sham animals, but this effect was reduced in the aortas of AB rats. Chronic treatments with losartan, captopril or amlodipine, but not with spironolactone, restored the Ang-(1-7)-induced aorta relaxation in AB rats. The coronary vasodilatation evoked by Ang-(1-7) in sham rats was blunted in hypertrophic rats. Only the treatment with losartan restored the coronary vasodilatory effect of Ang-(1-7) in AB rat hearts. These data support a beneficial vascular effect of an association of Ang-(1-7) and some antihypertensive drugs. Thus, this association may have potential as a new therapeutic strategy for cardiovascular diseases.

## Introduction

The renin-angiotensin system (RAS) is an important regulator of normal physiology and of the pathogenesis of cardiovascular disorders, including hypertension, pathological myocardial hypertrophy, heart failure, atherosclerosis, myocardial infarction and metabolic syndrome (1). Angiotensin (Ang) II and Ang-(1-7) are the main biologically active peptides of the RAS. Angiotensin converting enzyme 2 (ACE2) exerts an important role in this system since it can produce Ang-(1-7) through catalytic activity on Ang II (2). Ang-(1-7) produces many beneficial effects in the cardiovascular system, including vasodilatation, antihypertensive, antiarrhythmic and inhibition of pathological cardiac remodeling (3).

Ang-(1-7) induces vasodilation in several vascular beds, such as the aorta, mesenteric bed, renal artery and coronary arteries (4–6). The vascular effect of Ang-(1-7) is concentration-dependent, especially in coronary arteries. In nanomolar or micromolar concentrations, Ang-(1-7) did not affect the coronary circulation in isolated rat hearts (7). However, in picomolar concentration, Ang-(1-7) induces coronary vasodilation in isolated perfused mice hearts treated with losartan, an AT_1_ receptor antagonist (8). Recently, we have demonstrated an important coronary vasodilation elicited by picomolar concentration of Ang-(1-7) in isolated healthy rat hearts. This effect was absent in pressure overload-induced hypertrophic hearts. In addition, the vasorelaxation effect of Ang-(1-7) was impaired in aorta from pressure-overloaded rats. Interestingly, chronic treatment with losartan 1 mg·kg^-1^·day^-1^ restored the coronary vasodilation, but not aorta vasorelaxation in these rats (6). However, it is unknown whether higher doses of losartan could influence the effect of Ang-(1-7) in aorta or coronary arteries.

It is widely known that besides AT1 receptor blockers (ARBs), angiotensin-converting enzyme inhibitors (ACEIs), voltage-gated L-type calcium channel blockers, and mineralocorticoid receptor antagonists (MRAs) are also important pharmacological agents used for treatment of hypertension and other cardiovascular diseases (9–11). Furthermore, studies support the potential benefit of combination therapy in the prevention and treatment of these diseases (12). However, it is unknown whether these antihypertensive agents could also influence the vascular effects of the Ang-(1-7). Thus, in this study we investigated the influence of the ACEIs, ARBs, voltage-gated L-type calcium channel blockers and MRAs on the aortic and coronary effects of Ang-(1-7) in hypertrophied hearts.

## Material and Methods

### Animals

A total of 53 male Wistar rats weighing 250–300 g were used. The animals were provided by the animal facilities of the Universidade Federal de Goiás. All animals were kept in temperature-controlled rooms at 22±2°C with a 12/12 h light/dark cycle and had free access to water and food. All procedures were performed in accordance with institutional guidelines for the humane use of laboratory animals of our institution and were approved by the Ethics Committee for Animal Use of the Universidade Federal de Goiás (179/09).

### Blood pressure measurement

The rats were anesthetized with tribromoethanol (10 mg/kg of body weight), and a polyethylene catheter (PE-50) was inserted into the right carotid artery, tunneled under the skin and exteriorized at the neck. Mean arterial pressure (MAP) was measured in conscious rats 24 h after recovery from anesthesia. The data were recorded continuously with a PowerLab System device (ADInstruments, Australia).

### Hypertrophic heart rat model

Cardiac hypertrophy was induced by abdominal aortic banding (AB). After anesthesia was induced by tribromoethanol (10 mg/kg of body weight), a left laparotomy was performed, the descending aorta was isolated and a bent 21-gauge needle was placed next to the aorta. The suture was tied around the needle and the aorta at the level of abdominal aorta above the celiac artery. After ligation, the needle was quickly removed. In the sham group, age-matched animals underwent the same procedure without the placement of the aortic banding. The rats were euthanized 21 days after the AB or sham procedure. To evaluate cardiac hypertrophy, the left ventricular mass index (VMI) was calculated through the ratio between the left ventricular wet weight and tibia length.

### Chronic treatment

To evaluate the role of antihypertensive drugs on the effects of Ang-(1-7), some AB rats were treated with low doses of the AT_1_ receptor antagonist losartan, ACE inhibitor captopril, calcium channels blocker amlodipine, and mineralocorticoid receptor antagonist spironolactone for 21 days (5 mg/kg of body weight, per day), by gavage.

### Isolated aortic ring preparation

Isolated aortic rings were used to evaluate the effects of Ang-(1-7) on the thoracic aorta under pressure overload. Aortic rings (4 mm) from the descending thoracic aorta above the constriction or sham were placed in 10 mL organ baths at 37°C containing gassed (95% O_2_ and 5% CO_2_) Krebs-Henseleit solution with the following composition: 118.06 mM NaCl, 24.9 mM NaHCO_3_, 3.3 mM CaCl_2_·H_2_O, 4.6 mM KCl, 2.4 mM MgSO_4_·7H_2_O, 0.9 mM KH_2_PO_4_ and 11.1 mM glucose. The rings were maintained under a tension of 1.5 g for 1 h to equilibrate. Mechanical activity was recorded isometrically using a data acquisition system (DATAQ Instruments, USA). Endothelial integrity was considered by the ability of acetylcholine (Ach, 10^-5^ M) to induce more than 80% relaxation of vessels pre-contracted with phenylephrine (10^-7^ M). The effects of Ach (10^-9^–10^-5^ M) and Ang-(1-7) (10^-10^-10^-6^ M) were evaluated in aortic rings pre-constricted with phenylephrine (10^-7^ M). To evaluate the endothelium-independent relaxation, the endothelium was removed by rubbing the interior surface of the aorta. The success of this procedure was verified by the lack of aortic relaxation to Ach (10^-5^ M). Thereafter, the rings were contracted with phenylephrine (10^-7^ M) and exposed to sodium nitroprusside (10^-11^–10^-5^ M).

### Isolated heart preparation

The rats were decapitated 10–15 min after an intraperitoneal injection of 200 IU heparin. The thorax was opened and the heart was carefully dissected and perfused through the aortic stump with Krebs-Ringer solution containing 118.4 mM NaCl, 4.7 mM KCl, 1.2 mM KH_2_PO_4_, 1.2 mM MgSO_4_·7H_2_O, 1.25 mM CaCl_2_·2H_2_O, 11.7 mM glucose, and 26.5 mM NaHCO_3_. The perfusion flow was maintained constant (8–10 mL/min) at 37°C with constant oxygenation (5% CO_2_ and 95% O_2_). A balloon was inserted into the left ventricle through the left atrium for isovolumetric recordings of left ventricular pressures. Coronary perfusion was measured with a transducer connected to aortic cannula and coupled to a data-acquisition system (DATAQ Instruments). After a basal period (30–40 min), the hearts from sham and AB rats were perfused for an additional 15 minutes with Krebs-Ringer solution containing Ang-(1-7) (2×10^-11^ M).

### Drugs

Angiotensin-(1-7) was obtained from Bachem Inc. (USA). Acetylcholine, phenylephrine and sodium nitroprusside were obtained from Sigma Chemical Co. (USA), and losartan was obtained from Genix Pharmaceutical Industry (Brazil). Spironolactone was obtained from Gemini Industry and Commerce (Brazil), captopril was obtained from Pharma Nostra¯ (Brazil), and amlodipine was obtained from All Chemistry (Brazil).

### Data analysis

Results are reported as means±SE. Two-way ANOVA with Sidak multiple comparison or Dunnett's post-test were used to compare the curves obtained in isolated hearts and aortic ring preparation protocols. P<0.05 was considered to be statistically significant.

## Results

### Blood pressure and cardiac morphometric parameters

Blood pressure (BP) was measured in the carotid artery 21 days after abdominal banding. The MAP was significantly higher in AB rats compared with the sham group (107±1.7 *vs* 152±6.5 mmHg in AB, P<0.05). The treatment with low doses of losartan, captopril and amlodipine did not change the BP in AB rats (153±8.4, 131±2.1, and 152±7.3 mmHg, respectively, *vs* 152±6.5 mmHg in AB). Interestingly, the treatment with spironolactone reduced the BP in AB rats (152±6.5 *vs* 125±9.1 mmHg in Spi, P<0.05). To confirm the cardiac hypertrophy, morphometric analyses of the hearts were performed. Abdominal aortic banding induced a significant increase in VMI (0.224±0.007 *vs* 0.255±0.006 g/cm in AB, P<0.05). Losartan, amlodipine, and spironolactone did not alter the VMI (0.265±0.013, 0.275±0.010, and 0.249±0.014 g/cm, respectively, *vs* 0.255±0.006 g/cm in AB). However, the treatment with captopril reduced the pressure overload-induced left ventricular hypertrophy (0.255±0.006 *vs* 0.227±0.010 g/cm in Cap, P<0.05).

### Effects of Ang-(1-7) on isolated aortic rings from AB rats treated with losartan, captopril, amlodipine and spironolactone

**Figure 1:**
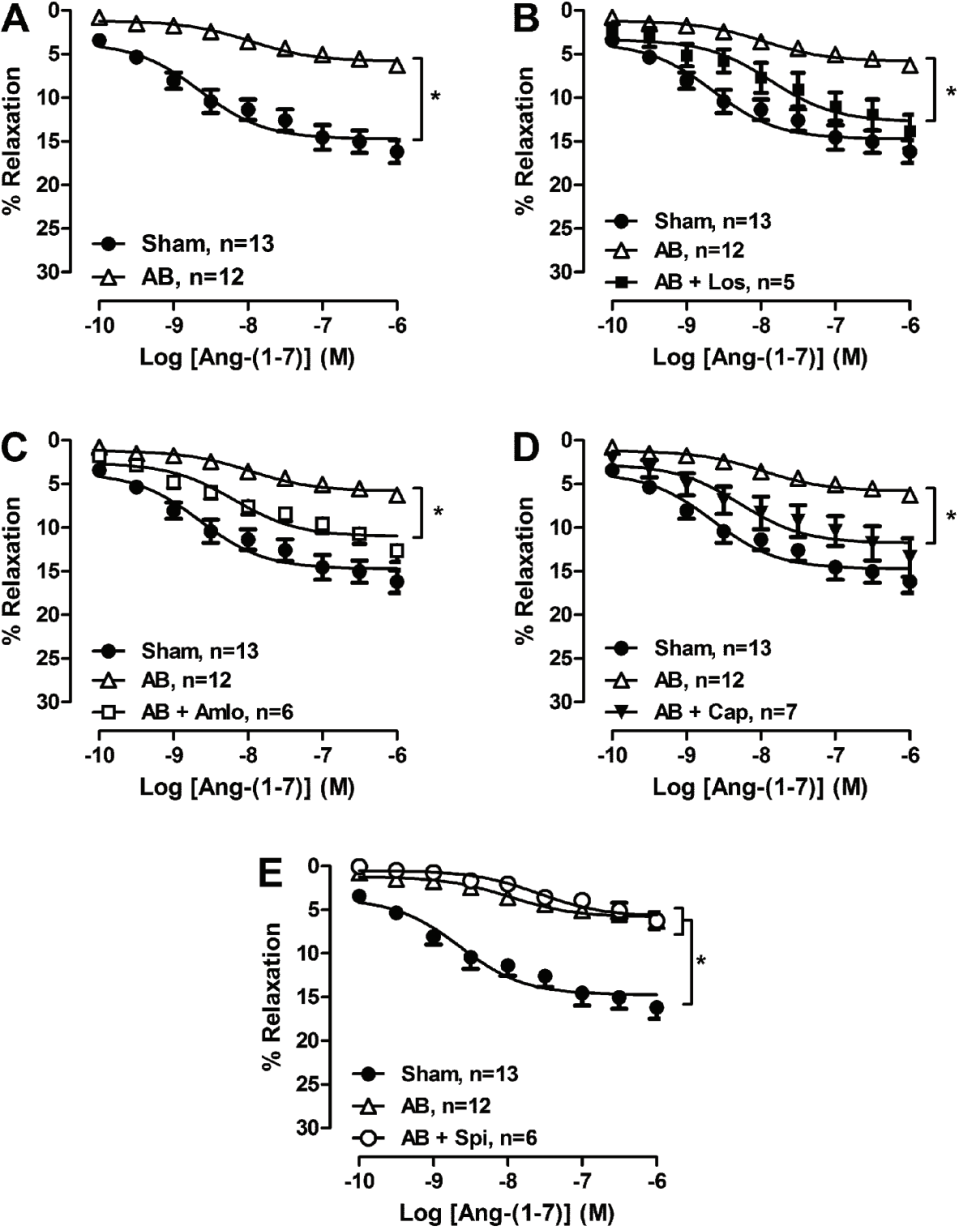
*A*, Effect of Ang-(1-7) on the vasorelaxation in aortic rings from rats that underwent aortic banding (AB). Effect of chronic treatment with (*B*) losartan, (*C*) amlodipine, (*D*) captopril or (*E*) spironolactone (5 mg/kg of body weight per day) in response to Ang-(1-7) in aortic rings from AB rats. Data are reported as means±SE. Los: losartan; Amlo: amlodipine; Cap: captopril; Spi: spironolactone. *P<0.05, two-way ANOVA followed by Sidak multiple comparison post-test.

As previously shown (6), Ang-(1-7) induced a significant relaxation in the aorta from sham animals and this effect was significantly reduced in the aortas of AB rats (Figure 1A). Differently from our previous study using 1 mg·kg^-1^·day^-1^(6), chronic treatment with losartan 5 mg·kg^-1^·day^-1^ restored the Ang-(1-7)-induced aorta relaxation in these rats (Figure 1B). At the same dose, captopril and amlodipine, but not spironolactone also restored the aorta relaxation promoted by Ang-(1-7) in AB aorta rats (Figure 1A-E).

### Effects of acetylcholine and sodium nitroprusside on isolated aortic rings from AB rats treated with losartan, captopril, amlodipine and spironolactone

**Figure 2:**
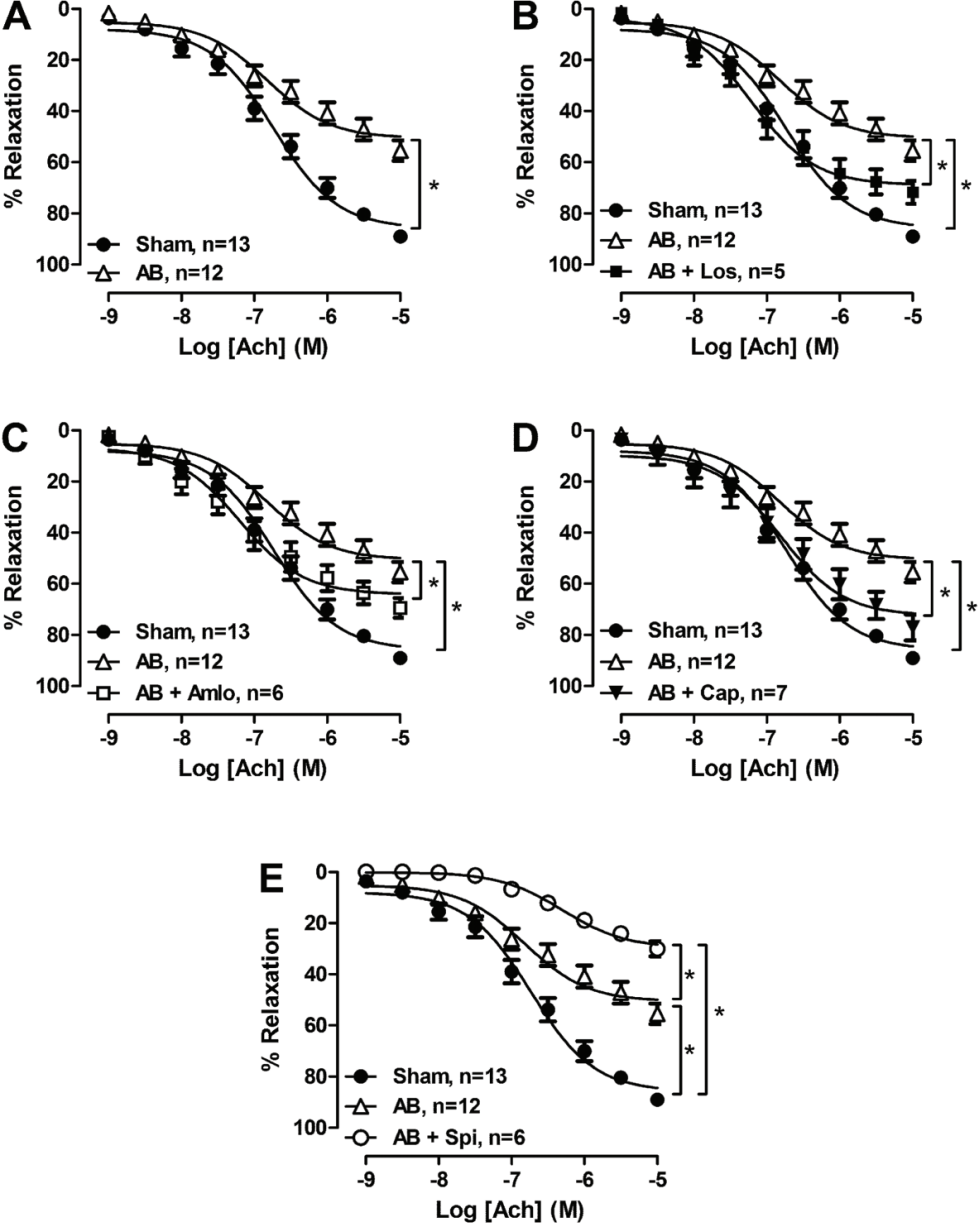
*A*, Effect of acetylcholine (Ach) on the vasorelaxation in aortic rings from rats that underwent aortic banding (AB). Effect of chronic treatment with (*B*) losartan, (*C*) amlodipine, (*D*) captopril or (*E*) spironolactone (5 mg/kg of body weight per day) in response to Ach in aortic rings from AB rats. Data are reported as means±SE. Los: losartan; Amlo: amlodipine; Cap: captopril; Spi: spironolactone. *P<0.05, two-way ANOVA followed by Sidak multiple comparison post-test.

The relaxation response to Ach was significantly reduced in aortic rings from AB rats (Figure 2A). Interestingly, losartan, amlodipine and captopril improved the Ach-induced relaxation in aortic rings from AB rats (Figure 2B–D). Unexpectedly, the relaxation induced by Ach was further reduced by treatment with spironolactone in AB rats (Figure 2E).

**Figure 3:**
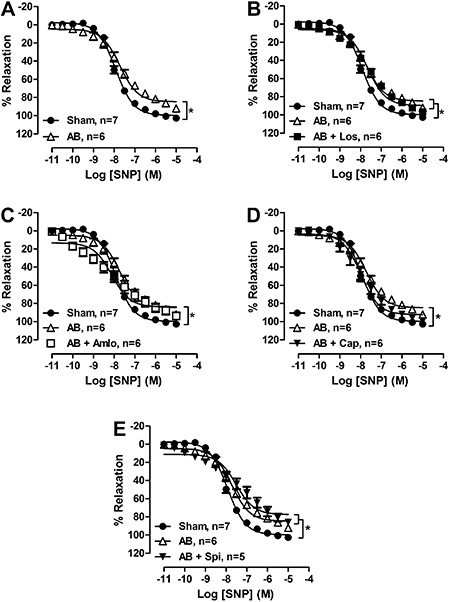
*A*, Effect of sodium nitroprusside (SNP) on the vasorelaxation in aortic rings from aortic banding (AB). Effect of chronic treatment with (*B*) losartan, (*C*) amlodipine, (*D*) captopril, or (*E)* spironolactone (5 mg/kg of body weight per day) in response to SNP in aortic rings without endothelium from AB rats. Data are reported as means±SE. Los: losartan; Amlo: amlodipine; Cap: captopril; Spi: spironolactone. *P<0.05, two-way ANOVA followed by Sidak multiple comparison post-test.

Indeed, the relaxation response to sodium nitroprusside was also impaired in aortic rings from AB rats (Figure 3A). None of antihypertensive drugs improved this effect (Figure 3B-E).

### Effects of Ang-(1-7) on coronary vasomotricity from AB rats treated with losartan, captopril, amlodipine, and spironolactone

**Figure 4:**
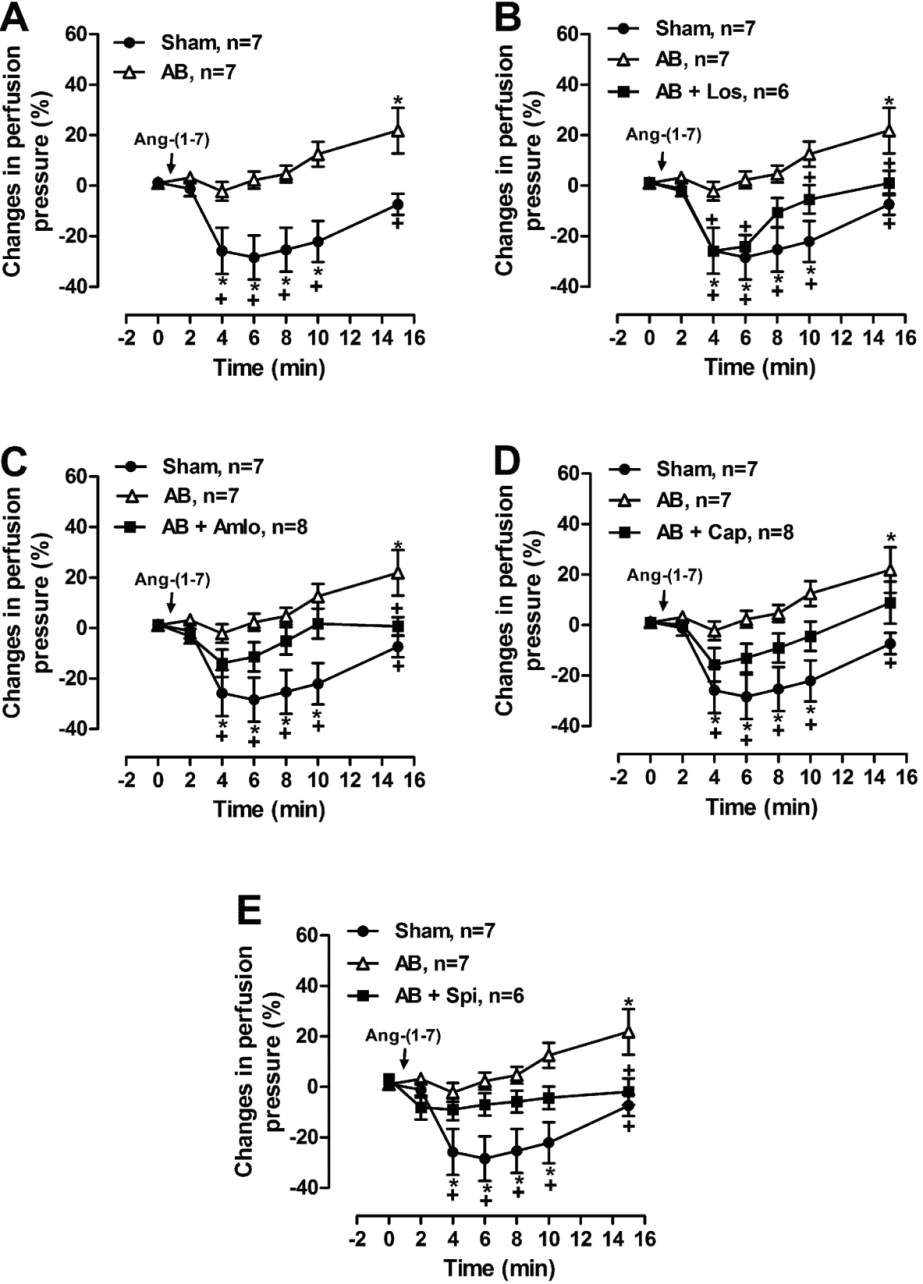
Effects of Ang-(1-7) (2×10^-11^ M) on coronary perfusion pressure in isolated perfused hearts from rats that underwent aortic banding (AB) and were (*A*) untreated or chronically treated with (*B*) losartan, (*C*) amlodipine, (*D*) captopril, or (*E*) spironolactone (5 mg·kg^-1^·day^-1^). Data are reported as means±SE. Los: losartan; Amlo: amlodipine; Cap: captopril; Spi: spironolactone. *P<0.05 compared with basal levels; ^+^P<0.05 between time points (two-way ANOVA followed by Dunnett’s to compare to basal levels; Sidak multiple comparison post-test to compare between time points).

The effects of Ang-(1-7) on the coronary vasomotricity were assessed in isolated Langendorff-perfused rat hearts. As observed in Figure 4A, Ang-(1-7) induced a significant coronary vasodilation in sham animals indicated by a decrease in perfusion pressure. This effect was not observed in coronary arteries of AB rats. Indeed, Ang-(1-7) induced an increase in the perfusion pressure in the heart of these animals. Chronic treatment with losartan in AB rats restored the coronary vasodilatory effect of Ang-(1-7) (Figure 4B). Differently, the treatment with captopril, amlodipine or spironolactone did not restore the vasodilator effect of Ang-(1-7) on hypertrophic hearts (Figure 4C–E). However, all antihypertensive drugs prevented the increase in the perfusion pressure induced by Ang-(1-7) in AB rat hearts.

## Discussion

The major findings of this study were that the aortic vasorelaxant effect evoked by Ang-(1-7) was completely restored by chronic treatment with losartan, captopril and amlodipine, but not with spironolactone. Only treatment with AT1 receptor antagonist restored the coronary vasodilatory effect of Ang-(1-7) on AB rat hearts. In addition, all antihypertensive drugs prevented the increase in the perfusion pressure induced by Ang-(1-7) in AB rat hearts.

Several studies have demonstrated that Ang-(1-7) is also able to promote a vasorelaxant effect in the aorta through mechanism involving Mas activation, and nitric oxide and prostaglandins release (13,14). Accordingly, we also observed this effect in the aorta from sham-operated rats. However, the vasorelaxant effect of Ang-(1-7) was abrogated in AB rats. Interestingly, the treatment with losartan at the dose of 5 mg·kg^-1^·day^-1^ restored the Ang-(1-7)-induced aorta relaxation in AB rats. In our previous study (6), the losartan treatment at the dose of 1 mg·kg^-1^·day^-1^ failed to restore the effect of Ang-(1-7). A number of studies have demonstrated endothelium dysfunction in hypertensive animal models (15). As Ang-(1-7) is an endothelium-dependent vasodilator (13), the beneficial effect of losartan may be related to the improvement of the endothelial function in the aorta independently from blood pressure changes, since losartan did not change the arterial blood pressure of the AB rats. In agreement, we also observed that losartan treatment improved the Ach-induced aorta relaxation.

We also demonstrated that captopril and amlodipine improved the vasorelaxant effect of Ang-(1-7) on aortic rings from AB rats. In contrast, captopril and amlodipine did not alter the blood pressure, but also improved the Ach-induced aorta relaxation. Indeed, none of antihypertensive drugs ameliorated the vasorelaxant effect of sodium nitroprusside. Thus, we can hypothesize that these antihypertensive drugs ameliorated the endothelium function in AB rats. The effect of the captopril and amlodipine in improving endothelial function has been demonstrated (16,17). In our study, the treatment with spironolactone did not restore the Ang-(1-7)-induced aorta relaxation. Furthermore, this antihypertensive further reduced the vasorelaxation evoked by Ach in aortic rings taken from AB rats. These findings indicate that beneficial effect of Ang-(1-7) on vascular actions is specific for some antihypertensive drugs.

Noteworthy, previous studies have reported an influence of antihypertensive drugs in ACE2-angiotensin-(1-7)-Mas axis. It was demonstrated that the treatment with ARBs, ACEIs, and Voltage-gated L-type calcium channel blockers augmented the plasma levels of Ang-(1-7) and ACE2 expression and activity (18-20). Thus, we cannot discard the possibility that the beneficial effect of antihypertensive drugs may be due to the improvement in the activity of the ACE2-angiotensin-(1-7)-Mas axis.

The effect of Ang-(1-7) on coronary vessels has been described in several studies (4,8). Recently, our group demonstrated that Ang-(1-7) induced coronary vasodilatation in healthy rat hearts, but this effect was completely blunted in pressure overload condition and restored with chronic treatment with losartan at the dose of 1 mg·kg^-1^·day^-1^(6). Here, the higher dose of losartan (5 mg·kg^-1^·day^-1^) also restored the Ang-(1-7)-induced coronary vasodilatation. However, the same dose (5 mg·kg^-1^·day^-1^) of captopril, amlodipine and spironolactone did not restore the effect of Ang-(1-7) on coronary vessels. Differently, Mueller et al. (10) observed in obese rats that MRA improves coronary endothelial-dependent vasodilation, and mineralocorticoid receptor activation impairs coronary endothelial-dependent vasodilation in healthy rats. The difference between our and that study might be due to the dose used. These observations indicated that the coronary bed has a greater responsiveness to AT1 blockade and suggest a direct effect of losartan on coronary vessels, i.e., independently of arterial pressure or hypertrophy. Accordingly, despite the antihypertrophic and antihypertensive effects of captopril and spironolactone, respectively, these drugs did not restore the effect of Ang-(1-7) on coronary vessels (21).

We can also hypothesize that the beneficial effect of losartan in improving coronary vasodilation induced by Ang-(1-7) may be due to the modulation in the dimerization between angiotensin receptors (22,23). Previous studies have described interactions of AT_1_ receptor with Mas (8,22). Kostenis et al. (22) have shown that AT_1_ and Mas can directly interact with each other leading to an altered response to Ang II in cultured mammalian cells. In that study, the functional role of this interaction was observed in Mas knockout mice, which showed enhanced Ang II-mediated vasoconstriction in mesenteric arteries (22). Thus, we can hypothesize that AT1 receptor blockade may potentiate the effects evocated by activation of the Mas receptor. Furthermore, since AT1 receptor presents constitutive activity (24), it is reasonable to consider that losartan, acting as an inverse agonist, may attenuate the vasoconstrictor pathway activity and consequently improve the vasodilatory effect of Ang-(1-7). However, further studies are necessary to better elucidate the mechanisms involved in the effects of the losartan in improving the coronary vasodilation induced by Ang-(1-7).

Strikingly, Ang-(1-7) induced an increase in the perfusion pressure in the hypertrophic hearts. Previous studies have reported the vasoconstrictor effect of Ang-(1-7) on isolated renal (25) and mesentery arteries (26). However, these studies have used micromolar concentrations of the Ang-(1-7). In both studies, the vasoconstrictor effect was completely blocked by acute pre-treatment with AT1 receptor antagonists. Differently, we used picomolar concentration. In addition, all of the chronic treatment with antihypertensive drugs prevented the coronary vasoconstriction induced by Ang-(1-7), making it unlikely that this effect could be mediated by AT1 receptor. Thus, further studies are necessary to better understand this finding.

In conclusion, these data support a beneficial vascular effect of an association of Ang-(1-7) and some antihypertensive drugs. Thus, this association may have potential as a new therapeutic strategy for cardiovascular diseases.
